# Supplementary material for: The interactome of intact mitochondria by cross-linking mass spectrometry provides evidence for coexisting respiratory supercomplexes
Source: Mol Cell Proteomics. 2017 Dec 8;17(2):216–32. doi: 10.1074/mcp.RA117.000470 (PMC5795388; doi:10.1074/mcp.RA117.000470)
Supplement: Supplemental Data [file supp_RA117.000470_133922_0_supp_23978_fzffwf.zip › spectra_annotation/mito_DR_spectra_annotation/3-1-14-1-6-1.pdf]

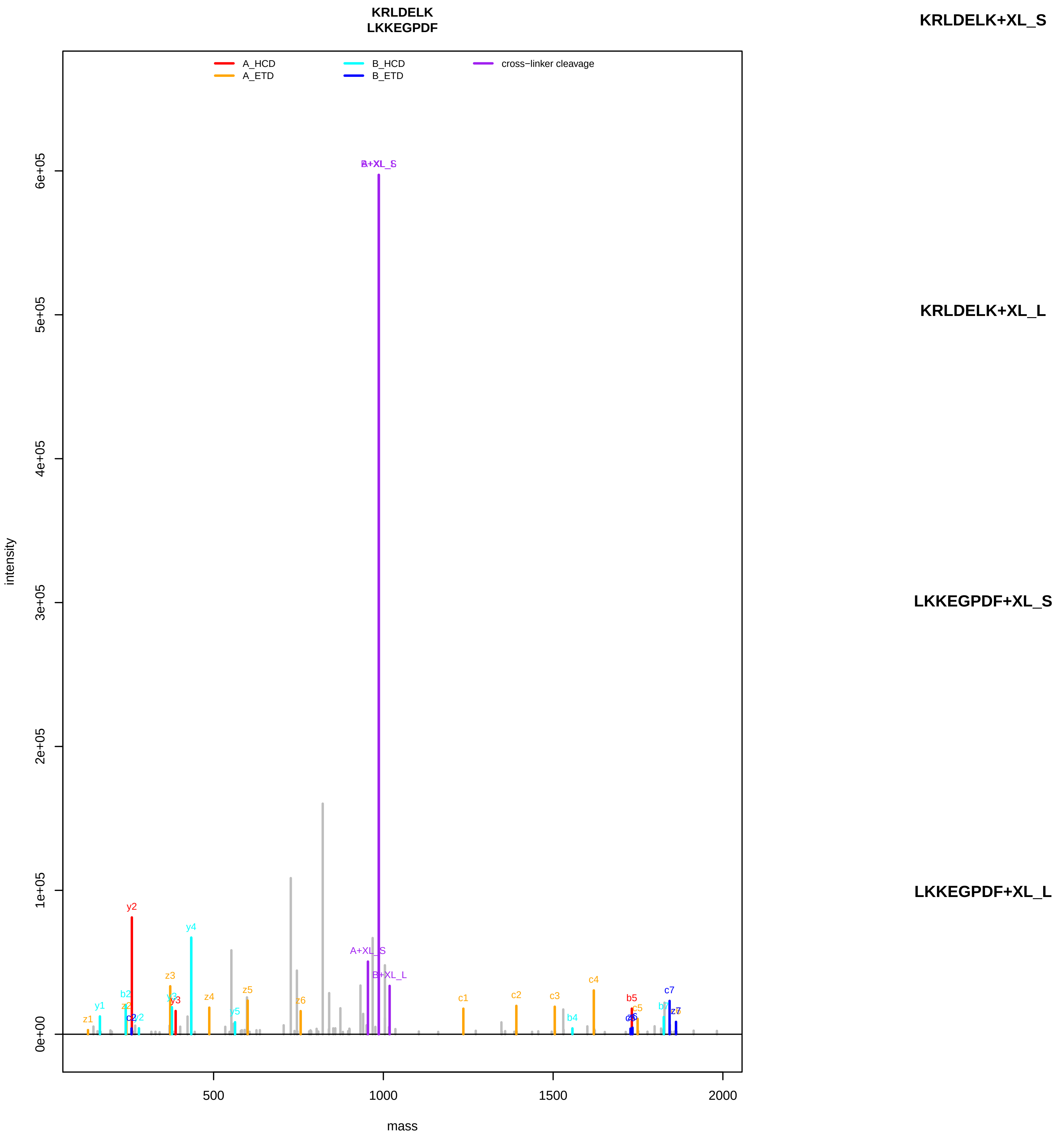
KRLDELK
LKKEGPDF
A_HCD
A_ETD
B_HCD
B_ETD
cross−linker cleavage
intensity
mass
0e+00
1e+05
2e+05
3e+05
4e+05
5e+05
6e+05
500
1000
1500
2000
y1
z1
b2
z2
y2
c2
y3
z3
y4
z4
y5
z5
z6
A+XL_S
B+XL_L
c1
c2
c3
c4
b4
b5
c5
c6
c7
z7
KRLDELK+XL_S
KRLDELK+XL_L
LKKEGPDF+XL_S
LKKEGPDF+XL_L
